# An Adaptive INS-Aided PLL Tracking Method for GNSS Receivers in Harsh Environments

**DOI:** 10.3390/s16020146

**Published:** 2016-01-23

**Authors:** Li Cong, Xin Li, Tian Jin, Song Yue, Rui Xue

**Affiliations:** School of Electronic and Information Engineering, Beihang University, 37 Xueyuan Road, Haidian District, Beijing 100191, China; congli_hlj@163.com (L.C.); lixin2015_xlm@163.com (X.L.); 1024Sam@buaa.edu.cn (S.Y.); xuerui@buaa.edu.cn (R.X.)

**Keywords:** GNSS, INS-aided PLL, high dynamic, signal attenuation, noise bandwidth, coherent integration time, adaptive adjustment

## Abstract

As the weak link in global navigation satellite system (GNSS) signal processing, the phase-locked loop (PLL) is easily influenced with frequent cycle slips and loss of lock as a result of higher vehicle dynamics and lower signal-to-noise ratios. With inertial navigation system (INS) aid, PLLs’ tracking performance can be improved. However, for harsh environments with high dynamics and signal attenuation, the traditional INS-aided PLL with fixed loop parameters has some limitations to improve the tracking adaptability. In this paper, an adaptive INS-aided PLL capable of adjusting its noise bandwidth and coherent integration time has been proposed. Through theoretical analysis, the relation between INS-aided PLL phase tracking error and carrier to noise density ratio (*C*/*N*_0_), vehicle dynamics, aiding information update time, noise bandwidth, and coherent integration time has been built. The relation formulae are used to choose the optimal integration time and bandwidth for a given application under the minimum tracking error criterion. Software and hardware simulation results verify the correctness of the theoretical analysis, and demonstrate that the adaptive tracking method can effectively improve the PLL tracking ability and integrated GNSS/INS navigation performance. For harsh environments, the tracking sensitivity is increased by 3 to 5 dB, velocity errors are decreased by 36% to 50% and position errors are decreased by 6% to 24% when compared with other INS-aided PLL methods.

## 1. Introduction

With the availability of accurate position, velocity and timing information that enables many applications we use in our daily lives, the global navigation satellite system (GNSS) has become nearly ubiquitous for both civil and military users [1,2]. However, compared with delay-locked loop (DLL), carrier tracking loop is commonly regarded as the weak link in GNSS signal processing. This fragility results from the larger carrier dynamics caused by line-of-sight (LOS) motion and the reduction of signal strength. In a highly dynamic environment, traditional phase-locked loop (PLL) can easily lose lock due to the rapid Doppler frequency changes. The GNSS signal is also easily attenuated below the normal tracking threshold due to the operating environment-induced interferences [3]. Usually, the most effective way to improve dynamic performance is to broaden the noise bandwidth and reduce the coherent integration time. However, a narrower noise bandwidth and longer integration time are required to decrease the thermal noise and improve the tracking sensitivity. For these two constraints, certain scenarios with high dynamic and signal attenuation will make it more difficult for GNSS signal to remain locked [4,5,6].

In general, the inertial navigation system (INS)-aided tracking method is an effective solution to this problem, since much of the vehicle dynamics is compensated by the inertial data, and the PLL bandwidth can be reduced [7,8]. The performance improvement of INS-aided GNSS tracking in highly dynamic applications with GNSS signal attenuation has been verified [9,10,11,12]. However, the loop noise bandwidth is constrained by the navigation solution error of the GNSS/INS and the receiver’s oscillator errors [13]. For civil and military vehicles with high dynamics, *i.e.*, urban unmanned aerial vehicles (UAVs), aeronautic/astronautic aircrafts and precision-guided weapons [3], when they are under harsh environment conditions such as severe ionospheric scintillation, radio frequency interference or jamming [13], the navigation solution error varies with signal dynamics and attenuation, which means that the INS-aided PLL with constant loop parameters cannot optimally accommodate the harsh environment. To further improve the tracking performance, the PLL capable of adjusting its bandwidth has been put forward to achieve more tracking robustness. For example, in [14], the adaptive bandwidth algorithm for the PLL without external aid works well in the environment with high carrier to noise density ratio (*C*/*N*_0_) and less random dynamic variations. Sun *et al.* [15] used a reduced inertial measurement unit (IMU) to aid a GPS receiver and designed an adaptive PLL loop filter capable of adjusting its bandwidth, which can improve both of the navigation performance and PLL tracking ability. A fuzzy logic controller (FLC) was also applied in [8] to choose an adaptive bandwidth for INS-aided PLL, and the improvement of tracking performance was demonstrated by simulation results.

In addition to the noise bandwidth, the coherent integration time of the tracking loop is also one of the major factors impacting tracking performance [10,16,17], especially for harsh environments. The changing integration time implies a need to adjust the loop update rate. By analyzing the Kalman filter-based carrier-phase tracking loop, O’Driscoll [6] drew the conclusion that there is a trade-off between epoch-by-epoch detectability and the deleterious effect of reducing the loop update rate, and proposed a method to choose the optimal integration time for the carrier loop without INS aid based on a given condition. Hence, an adaptive INS-aided PLL tracking method capable of adjusting both its noise bandwidth and integration time could achieve better performance than the method of only adjusting the noise bandwidth in the harsh environment. However, in practical application, since the update rates of GNSS and INS are generally asynchronous, the choices of different integration time and aiding information update time will result in different tracking errors and the integration time adjustment will also increase the complexity of the tracking loop implementation, therefore we need to consider the influences of various factors on the INS-aided tracking loop to design an adaptive tracking method.

This paper focuses on an INS-aided PLL tracking method for GNSS receiver in harsh environments with high dynamics and signal attenuation. The PLL is aided with delta Doppler from the integrated system. Based on a comprehensive theoretical analysis of the relation between INS-aided PLL tracking error and *C*/*N*_0_, vehicle dynamics, aiding information update time, noise bandwidth and integration time, a method to choose the optimal integration time, noise bandwidth for a given application under the minimum tracking error criterion has been proposed. By means of the adjustments of both integration time and noise bandwidth, the adaptive ability of the INS-aided GNSS carrier tracking loop for the harsh environment can be effectively improved.

The rest of this paper is organized as follows: Section 2 gives an overview of the principle of delta Doppler-aided PLL. In Section 3, the main error sources of the INS-aided PLL are analyzed and the phase errors are derived by the discrete-time domain approach. Section 4 describes the method for choosing the optimal noise bandwidth and integration time. In Section 5 and Section 6, software and hardware simulations are conducted to test the adaptive tracking method. In Section 7, the method is evaluated through a field test using a vehicle driven in urban environments. The final conclusions of this paper are given in Section 8.

## 2. The Principle of Delta Doppler Aided PLL

**Figure 1:**
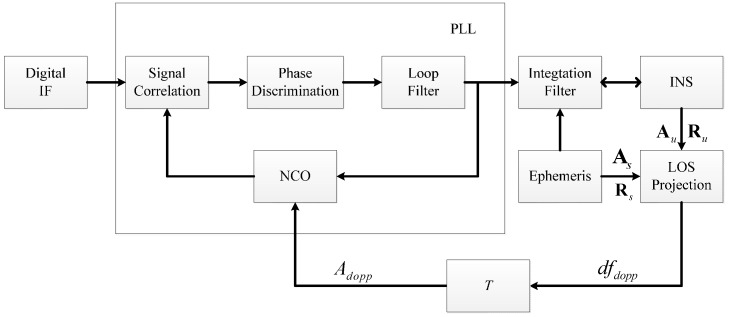
The delta Doppler aided PLL architecture.

Usually, two kinds of INS information are used to aid the carrier loop: Doppler derived from the integration filter [4] and delta Doppler over integration time [18]. For a second-order carrier loop, there is no steady-state error when tracking the velocity under ideal conditions, while there will be a steady-state error under acceleration. Velocity and acceleration correspond to Doppler frequency and Doppler rate, respectively. As a matter of fact, the performance of the carrier loop is mainly determined by the Doppler rate and less so by the Doppler frequency. Therefore, we use the delta Doppler derived from INS as the aiding information. The delta Doppler aided PLL architecture can be described as in Figure 1.

The Doppler rate $df_{dopp}$ can be obtained as:

(1) $$df_{dopp}=-\frac{1}{\lambda }\cdot \left(A_{u}-A_{s}\right)\cdot \frac{\left(R_{u}-R_{s}\right)}{\left|R_{u}-R_{s}\right|}$$

where λ is the wavelength of the carrier, **A***_u_*, **A***_s_* are three-dimensional acceleration vectors of receiver and satellite, respectively, and **R***_u_*, **R***_s_* are three-dimensional position vectors of receiver and satellite, respectively. With the integration time *T* of the PLL, the aiding information, *i.e.,* delta Doppler, can be expressed as the product of Doppler rate and integration time as follows:

(2) $$A_{dopp}=df_{dopp}\cdot T$$

## 3. Tracking Error Analysis of Delta Doppler Aided PLL

In the case of harsh environments with high dynamics and signal attenuation, the dynamic stress error and thermal noise are the most critical INS-aided PLL error sources, and oscillator phase error will also influence the tracking performance [19]. Here we analyze these three major error sources. In order to accurately analyze the effect of the coherent integration time on the tracking error of INS-aided PLL, it is necessary to consider the discrete nature of the loop [6].

### 3.1. Dynamic Stress Error

In [20], the traditional PLL tracking error in discrete-time domain has been analyzed, so we can obtain the dynamic stress error of traditional second-order PLL by analyzing the steady-state phase error of the loop as:

(3) $$E[\Psi_{ss}]=\frac{a}{\omega_{n}^{2}}(1+\zeta \omega_{n}T+\frac{1}{4}{\left(\omega_{n}T\right)}^{2})$$

where E[⋅] is mathematical expectation function, **Ψ***_ss_* is the steady-state of the phase error, *a* is the line-of-sight (LOS) vehicle acceleration, *T* is the integration time of the PLL, ζ is the damping factor, and ω*_n_* is characteristic frequency of the loop. The noise bandwidth *B_n_* is related with ω*_n_* and ζ as follows:

(4) $$B_{n}=\frac{\omega_{n}}{2}(\zeta +\frac{1}{4\zeta })$$

**Figure 2:**
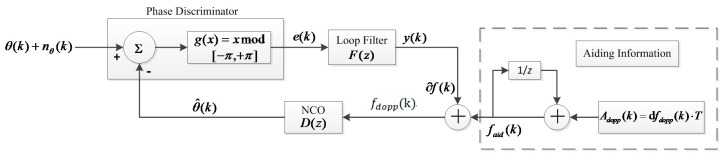
Structure of delta Doppler-aided PLL in discrete-time domain.

From Equation (3) we know that dynamic stress is proportional to the LOS acceleration, and is inversely proportional to the square of the bandwidth. The structure of delta Doppler-aided PLL in the discrete-time domain is illustrated in Figure 2. The process of delta Doppler-aided tracking is actually the integral process of aiding information *A_dopp_*(*k*).

Due to the random nature of the input phase noise, the output of the discriminator should be a random process as well. From Figure 2, we have the following equations:

(5) $$e(k)=g[\psi (k)]+n_{\theta }(k),e(k)\in [-\pi ,\pi ]$$

(6) $$\psi (k)=\theta (k)-\hat{\theta }(k),\Psi (k)=g[\psi (k)]\in [-\pi ,\pi ]$$

(7) $$\hat{\theta }\left(z\right)=D\left(z\right)f_{dopp}(z)$$

(8) $$f_{dopp}(z)=F(z)\Psi \left(z\right)+\frac{1}{1-z^{-1}}A_{dopp}\left(z\right)$$

where *e*(*k*) is the output of the phase discriminator, θ(k) and $\hat{\theta }(k)$ are the phases of the incoming signal and the local NCO signal at the center of the interval, respectively, ψ(*k*) is the phase tracking error due to a noise-free incoming signal, n_θ_(*k*) is the phase disturbance due to the input noise, g[⋅] is a characteristic function of the phase discriminator, Ψ(*k*) is the restricted phase error, the NCO transfer function is *D*(*z*) = *T*·*z*^−1^/(1 − *z*^−1^), *y*(*k*) is the output of the loop filter at the end of the *kth* correlation interval, and *F*(*z*) is transfer function of second-order loop filter that can be written as:

(9) $$F(z)=\frac{(G_{1}+G_{2})-G_{1}z^{-1}}{T\cdot (1-z^{-1})}$$

where *G*_1_ and *G*_2_ denote the parameters of the loop filter in discrete-time domain which can be expressed as:

(10) $$\{\begin{array}{c} G_{1}=\frac{1}{K}\frac{8\zeta \omega_{n}T}{4+4\zeta \omega_{n}T+{\left(\omega_{n}T\right)}^{2}}\\ G_{2}=\frac{1}{K}\frac{4{\left(\omega_{n}T\right)}^{2}}{4+4\zeta \omega_{n}T+{\left(\omega_{n}T\right)}^{2}} \end{array}$$

By substituting Equations (7)–(9) into Equation (6), we obtain:

(11) $$\Psi \left(z\right)=\frac{\left(1-z^{-1})\theta (z\right)}{{\left(1-z^{-1}\right)}^{2}+\left(G_{1}+G_{2}\right)z^{-1}-G_{1}z^{-2}}-\frac{A_{dopp}(z)Tz^{-1}}{{\left(1-z^{-1}\right)}^{2}+\left(G_{1}+G_{2}\right)z^{-1}-G_{1}z^{-2}}$$

Using the analytical method of [20], we can obtain the differential equation of the phase tracking error of INS-aided PLL, which can be expressed as:

(12) $$\begin{array}{l} \Psi \left(k\right)-2\Psi \left(k-1\right)+\Psi \left(k-2\right)=\theta \left(k\right)-2\theta \left(k-1\right)+\theta \left(k-2\right)\\ -\left(G_{1}+G_{2}\right)\Psi \left(k-1\right)+G_{1}\Psi \left(k-2\right)-A_{dopp}\left(k-1\right)T \end{array}$$

Under ideal condition, the aiding Doppler rate *df_dopp_*(*k*) is equal to second-order derivative of incoming signal phase $\ddot{\theta }\left(k\right)$. However, there will be an aiding delay Δ*t* caused by the asynchronous update rates of GNSS and INS, which is related to the aiding information update time *T_f_* and integration time *T* [21]. When the LOS dynamic is the first-order or second-order change of phase, as $\ddot{\theta }\left(k\right)$ is a constant value, the aiding delay will not affect the dynamic stress error, so the dynamic stress of the second-order loop caused by the acceleration can be removed. 

When there is a jerk in the LOS dynamic, we have:

(13) $$\theta \left(k\right)=\frac{1}{6}pk^{3}+\frac{1}{2}ak^{2}+vk+\Theta ,k\geq 0$$

where Θ is the initial phase, *v* is the phase change rate, *a* and *p* are the acceleration and jerk of the phase change, respectively. With the existence of the jerk *p*, the aiding Doppler rate will have the error caused by the aiding delay, so we have:

(14) $$df_{dopp}\left(k-1\right)=\ddot{\theta }\left(k-1\right)-p\Delta t$$

In addition, because of the calculation error of satellite acceleration Δ*a_kSat_* and the LOS acceleration error Δ*a_kINS_* caused by the error of INS, the aiding Doppler rate can be expressed as:

(15) $$\begin{array}{l} df_{dopp}\left(k-1\right)=\ddot{\theta }\left(k-1\right)-\left(p\Delta t+\Delta a_{kSat}+\Delta a_{kINS}\right)\\ =\left\{\theta \left(k\right)-2\theta \left(k-1\right)+\theta \left(k-2\right)\right\}/T^{2}-\left(p\Delta t+\Delta a_{kSat}+\Delta a_{kINS}\right) \end{array}$$

Then the aiding information *A_dopp_*(*k* − 1) can be obtained as:

(16) $$A_{dopp}\left(k-1\right)=pT^{2}(k-1)+aT-(p\Delta t+\Delta a_{kSat}+\Delta a_{kINS})T$$

Substitute the Equations (16) and (13) into Equation (12), and according to Equation (3), the dynamic stress error of delta Doppler aided PLL can be derived as:

(17) $$E[\Psi_{ss}]=\frac{\Delta a_{kSat}+\Delta a_{kINS}+p\Delta t}{\omega_{n}^{2}}(1+\zeta \omega_{n}T+\frac{1}{4}{\left(\omega_{n}T\right)}^{2})$$

Δ*a_kSat_* is usually small enough to be ignored [14], and Δ*a_kINS_* can be expressed as [19]:

(18) $$\Delta a_{kINS}=\delta f_{ib}^{b}\cos\left(\omega_{s}t_{c}\right)+\delta \omega_{ib}^{b}g\frac{\sin\left(\omega_{s}t_{c}\right)}{\omega_{s}}$$

where $\delta \omega_{ib}^{b}$ and $\delta f_{ib}^{b}$ are the total angular rate and force error in the body frame, *g* is the acceleration due to gravity, ω*_s_* is the Schuler frequency and *t_c_* is time with no INS error corrections.

**Figure 3:**
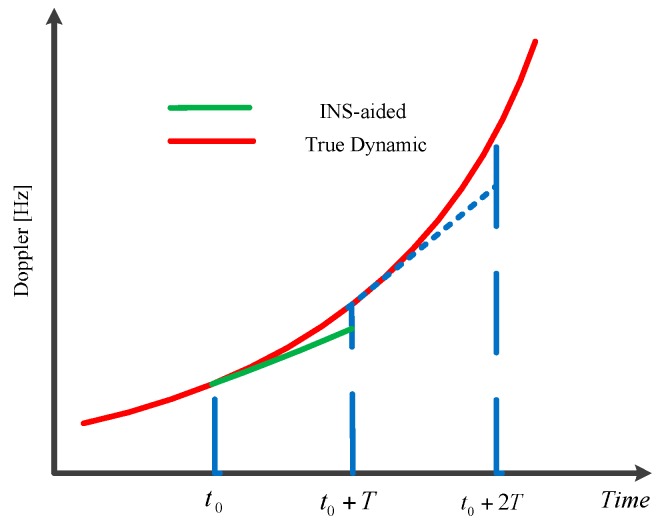
Aiding delay error with a jerk dynamic.

The aiding delay Δ*t* can be analyzed as follows: the aiding delay error of Doppler with a jerk dynamic is illustrated in Figure 3, which gives a way to determine the value of Δ*t* as well. When the integration time is equal to the aiding information update time (*T* = *T_f_*), we usually use the acceleration at the time *t*_0_ to aid the loop over the interval from *t*_0_ to *t*_0_ + *T*.

Then we will obtain an average acceleration over the integration interval as (note that herein the update of NCO includes the update of the loop and the update of the aiding information):

(19) $$a_{M}=a_{0}+\frac{T}{2}p$$

where *a*_0_ is the acceleration at the time *t*_0_.

With the aiding acceleration *a_f_* = *a*_0_, the equivalent aiding delay can be derived as:

(20) $$\Delta t=\frac{a_{M}-a_{f}}{p}=\frac{T}{2}$$

The equation above is obtained under the condition of *T* = *T_f_*, let’s assume that Δ*t* is a weighted result of integration time and aiding information update timewith weights *k*_1_ and *k*_2_, and then let:

(21) $$\Delta t=k_{1}\cdot T_{f}+k_{2}\cdot T$$

Decreasing the integration time (loop update time) or the aiding information update time can improve the dynamic performance of INS-aided PLL. According to the selection of the loop update time and aiding information update time, the weights *k*_1_ and *k*_2_ should satisfy following conditions:

(22) $$\{\begin{array}{cc} k_{1}+k_{2}=\frac{1}{2} & T_{f}=T\\ k_{1}\cdot T_{f}+k_{2}\cdot T<\frac{T}{2} & T_{f}<T\\ k_{1}\cdot T_{f}+k_{2}\cdot T<\frac{T_{f}}{2} & T<T_{f} \end{array}$$

From Equation (22), we can find symmetry between integration time and update time of aiding information. With little empirical evidence available, let’s assume:

(23) $$k_{1}=k_{2}=\frac{1}{4}$$

Then we have:

(24) $$\Delta t=\frac{1}{4}(T_{f}+T)$$

Therefore, the dynamic stress error of INS-aided PLL can be expressed as:

(25) $$E[\Psi_{ss}]=\frac{\Delta a_{kINS}+\Delta a_{kSat}+0.25p\cdot (T_{f}+T)}{w_{n}^{2}}\cdot \left[1+\zeta w_{n}T+\frac{1}{4}{\left(w_{n}T\right)}^{2}\right]$$

### 3.2. Thermal Noise Error

The variance of the steady-state phase tracking error $\Psi_{ss}^{T}$ due to the input thermal noise $n_{\theta }^{T}$ is given as [20]:

(26) $$\mathrm{var}[\Psi_{ss}^{T}]=2B_{n}T(1+\frac{64\zeta^{4}+16\zeta^{2}+16\zeta^{2}B_{n}T}{\left(16\zeta^{4}+8\zeta^{2}+1)(4\zeta^{2}+1\right)}B_{n}T)\mathrm{var}[n_{\theta }^{T}]$$

where var[⋅] is the variance function. The variance of thermal noise is related to *C*/*N*_0_ and integration time *T*, which can be expressed as:

(27) $$\mathrm{var}[n_{\theta }^{T}]=\frac{1}{2TC/N_{0}}\left(1+\frac{1}{2TC/N_{0}}\right)$$

Define:

(28) $$c_{2}=2B_{n}T(1+\frac{64\zeta^{4}+16\zeta^{2}+16\zeta^{2}B_{n}T}{\left(16\zeta^{4}+8\zeta^{2}+1)(4\zeta^{2}+1\right)}B_{n}T)$$

Then, $\mathrm{var}[\Psi_{ss}^{T}]$ can be expressed as:

(29) $$\mathrm{var}[\Psi_{ss}^{T}]=c_{2}\mathrm{var}[n_{\theta }^{T}]$$

### 3.3. Oscillator Phase Error

For second-order PLL, the variance of oscillator phase error $n_{\theta }^{osc}$ can be expressed as [22]:

(30) $$\mathrm{var}[n_{\theta }^{osc}]=2\pi^{2}{f_{L}}^{2}[\frac{\pi^{2}h_{-2}}{\sqrt{2}{\omega_{n}}^{3}}+\frac{\pi h_{-1}}{4{\omega_{n}}^{2}}+\frac{h_{0}}{4\sqrt{2}\omega_{n}}]$$

where the oscillator parameters *h*_–2_[*s*^–1^], *h*_–1_[*dimensionless*] and *h*_0_[*s*] are related to the Allan deviation and *f_L_* is the carrier frequency.

Oscillator phase error is a reflection of oscillator’s characteristics, it can be considered as an input noise of the loop. Then the variance of the steady-state phase tracking error $\Psi_{ss}^{osc}$ due to the oscillator phase error can be expressed as:

(31) $$\mathrm{var}[\Psi_{ss}^{osc}]=c_{2}\mathrm{var}[n_{\theta }^{osc}]$$

### 3.4. Stable Tracking Criterion of INS-Aided PLL

According to Equation (5), we can get the output of the phase discriminator *e*(*k*) = Ψ(*k*) + *n*_θ_(*k*), then the mean of the steady-state output of the phase discriminator *e_ss_* is the dynamic stress error, which can be expressed as:

(32) $$E[e_{ss}]=E[\Psi_{ss}]$$

And the variance of the steady-state output of the phase discriminator due to the input noise $n_{\theta }=n_{\theta }^{T}+n_{\theta }^{osc}$ can be calculated as:

(33) $$\begin{array}{l} \mathrm{var}[e_{ss}]=\mathrm{var}[\Psi_{ss}]+\mathrm{var}[n_{\theta }]=\mathrm{var}[\Psi_{ss}^{T}]+\mathrm{var}[\Psi_{ss}^{osc}]+\mathrm{var}[n_{\theta }^{T}]+\mathrm{var}[n_{\theta }^{osc}]\\ =(1+c_{2})(\mathrm{var}[n_{\theta }^{T}]+\mathrm{var}[n_{\theta }^{osc}]) \end{array}$$

In order to ensure stable tracking, we select the phase tracking error of discriminator output as the criterion which can be expressed as:

(34) $$\sigma_{pd}=\sqrt{\mathrm{var}[e_{ss}]}+\frac{1}{3}E[e_{ss}]\leq 15^{\circ }$$

## 4. Adaptive Adjustments of Integration Time and Bandwidth of INS-Aided PLL

From Equation (34), we can find the relation between the phase tracking error of discriminator output and aiding information update time, integration time, noise bandwidth, *C*/*N*_0_, vehicle dynamics. Usually, a shorter INS prediction update time is selected as the aiding information update timeto improve the dynamic performance of INS-aided PLL [19]. Thus, for a certain *C*/*N*_0_ and vehicle dynamics condition, only a two-dimensional search of noise bandwidth and integration time is needed to find the minimum tracking error of discriminator output in theory, meanwhile the corresponding noise bandwidth and integration time are the optimal ones, respectively.

According to Equations (18) and (30), Δ*a_kINS_* and $\mathrm{var}[n_{\theta }^{osc}]$ can be obtained. And the method is proposed for adaptive adjustments of *B_n_* and *T* as below in Algorithm 1.

**Figure 4:**
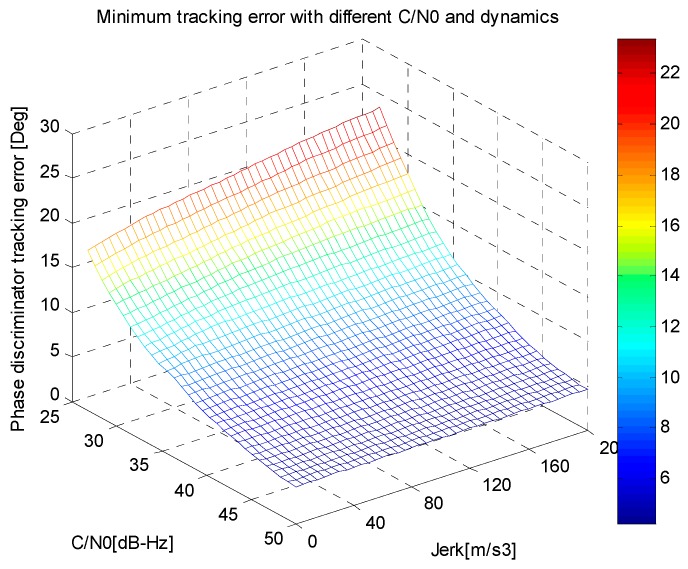
The minimum phase tracking error for different dynamics and *C*/*N*_0_.

**Figure 5:**
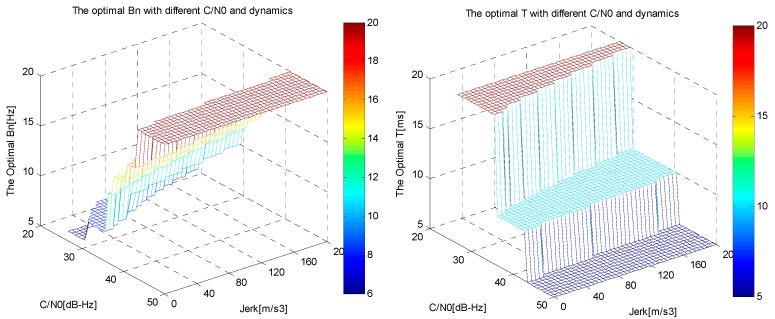
Optimal choices of *B_n_* (**left**) and *T* (**right**) for different dynamic and *C*/*N*_0_.

**Algorithm 1.** The adaptive method for choosing the optimal noise bandwidth and integration time.
**Input:**The variance of oscillator phase error, $\mathrm{var}[n_{\theta }^{osc}]$;The LOS acceleration error, Δ*a_kINS_*;Carrier to noise density ratio, *C*/*N*_0_;The output of the phase discriminator, *e*(*k*);The bandwidth and integration time of the last epoch, *B_n_*’ and *T*’;The choices of integration time: 1ms, 5ms, 10ms and 20 ms;A range of bandwidth from 1 Hz to 20 Hz with an interval of 2 Hz;
**Output:**
The optimal bandwidth and integration time under certain vehicle dynamic and carrier to noise ratio, [*B_n_* ,*T*];1:Considering the real-time performance and accuracy, a one-second sliding window is used to evaluate the mean of the PLL discriminators output *E*[Ψ*_ss_*] using Equation (32). Then the residual LOS acceleration error can be estimated;2:According to *E*[Ψ*_ss_*], Δ*a_kINS_*, $\mathrm{var}[n_{\theta }^{osc}]$, *B_n_*’ and *T*’, *p* can be calculated by Equation (17);3:Based on the calculated *C*/*N*_0_, Δ*a_kINS_*, $\mathrm{var}[n_{\theta }^{osc}]$ and *p*, we take different integration time and bandwidth as referred above, and calculate the phase tracking error with different integration time and bandwidth according to Equation (34);4:When the tracking error is the minimum, select the optimal bandwidth and integration time, [*B_n_* ,*T*];5:**return** [*B_n_*, *T*];


According to Equation (34), the minimum phase tracking error of the discriminator output for different LOS jerk and *C*/*N*_0_ is interpreted in Figure 4, while the corresponding optimal choices of *B_n_* and *T* are illustrated in Figure 5, where the carrier frequency is GPS L1 with a value of 1575.42 MHz, the aiding information update timeis *T_f_* = 10 ms (the output frequency of the inertial sensor used here is 100 Hz), the damping factor of second-order is ζ = 0.707, the parameters of a temperature controlled crystal oscillator (TCXO) are *h*_0_ = 1.0 × 10^−21^, *h*_–2_ = 1.0 × 10^−20^, and the biases of gyro and accelerometer are $\delta \omega_{ib}^{b}=10^{{}^{\circ}}/h$ and $\delta f_{ib}^{b}=1\;\text{mg}$, respectively.

As can be seen in Figure 4 and Figure 5, the minimum phase tracking error increases with lower *C*/*N*_0_ and higher vehicle LOS jerk. The optimal choices of bandwidth and integration time vary with *C*/*N*_0_ and vehicle dynamicd. For a certain *C*/*N*_0_, increasing the vehicle dynamics will increase the optimal bandwidth, but decrease the optimal integration time, and under certain dynamics, decreasing *C*/*N*_0_ will increase the optimal integration time, but decrease the optimal bandwidth. Hence, in order to obtain the minimum tracking error, we should choose appropriate integration time and bandwidth for certain vehicle LOS jerk and *C*/*N*_0_.

## 5. Software Simulation

**Figure 6:**
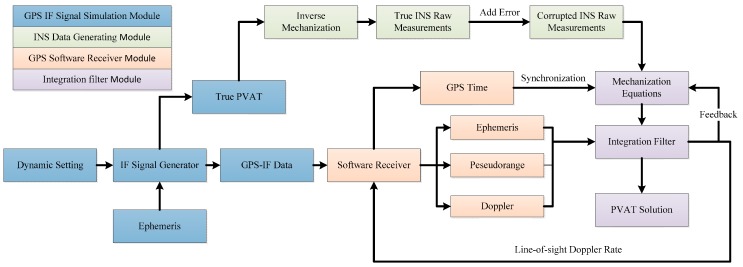
The block diagram of software simulation platform.

A software simulation platform was developed for the performance tests and analysis of the adaptive tracking method. The simulation system block diagram is shown in Figure 6. It includes four parts: GPS intermediate frequency (IF) signal simulation module, INS data generating module, GPS software receiver module and integration filter module.

To assess the performance advantages of the proposed adaptive tracking method, several tests are conducted. First, we verify the theoretical dynamic stress error analysis results by comparing them to simulated ones. Then we compare the actual phase tracking error of the discriminator output with the theoretically calculated one. Finally, through comparison with the traditional tracking method, the performance improvements of the adaptive tracking method can be verified.

### 5.1. Validation of Dynamic Stress Error

As shown in Equation (25), the dynamic stress error is related to the residual vehicle dynamic, integration time, noise bandwidth and update time of aiding information. Therefore, we can compare the mean of the output of phase discriminator with the theoretical value by setting different vehicle dynamic, noise bandwidth and integration time. 

**Figure 7:**
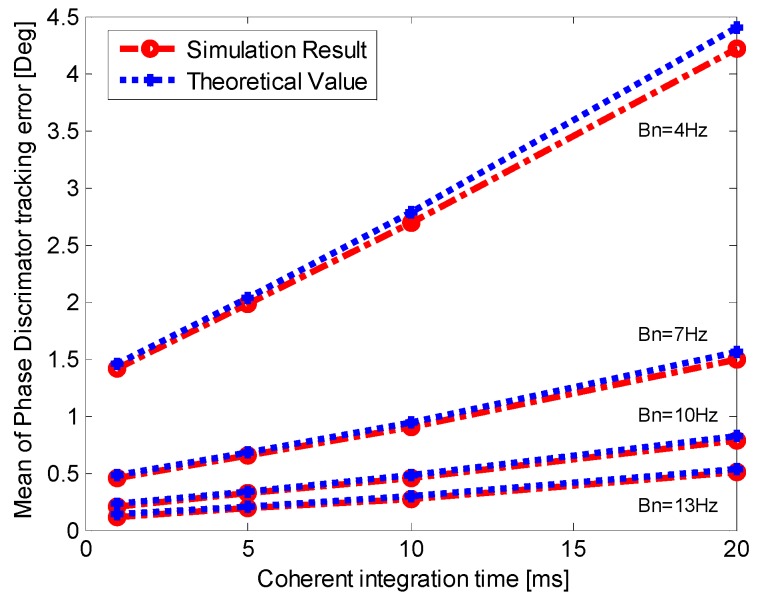
Mean of the output of PD (phase discriminator) when *p* = 50 m/s^3^.

**Figure 8:**
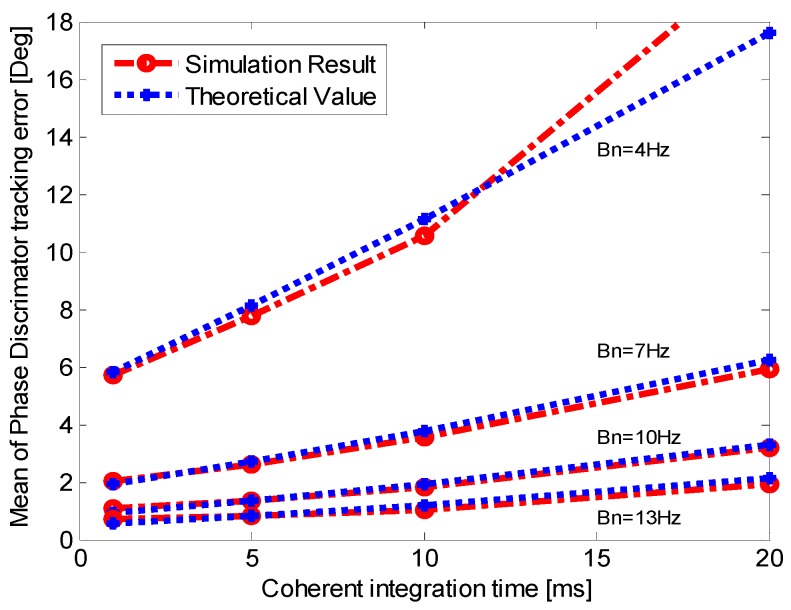
Mean of the output of PD when *p* = 200 m/s^3^.

To analyze the dynamic stress error, single-channel simulations are conducted with *C*/*N*_0_ = 50 dB-Hz and LOS jerk *p* = 50 m/s^3^. The aiding information update timeis set as 10 ms which is the INS prediction update time. The mean of the output of phase discriminator for different bandwidth and integration time is shown in Figure 7. The result when the jerk is increased to 200 m/s^3^ is shown in Figure 8. In Figure 8, when *T* is 20 ms and the noise bandwidth is 4 Hz, the theoretical value exceeds the stable tracking threshold, so it is not taken into consideration. Figure 7 and Figure 8 demonstrate that the dynamic stress error of INS-aided PLL increases with the vehicle LOS jerk, and it also increases with the narrower noise bandwidth and longer integration time. The simulation results fit well with the theoretically calculated ones, and show that the theoretical analysis of the dynamic stress error is correct.

### 5.2. Validation of Minimum Tracking Error

**Figure 9:**
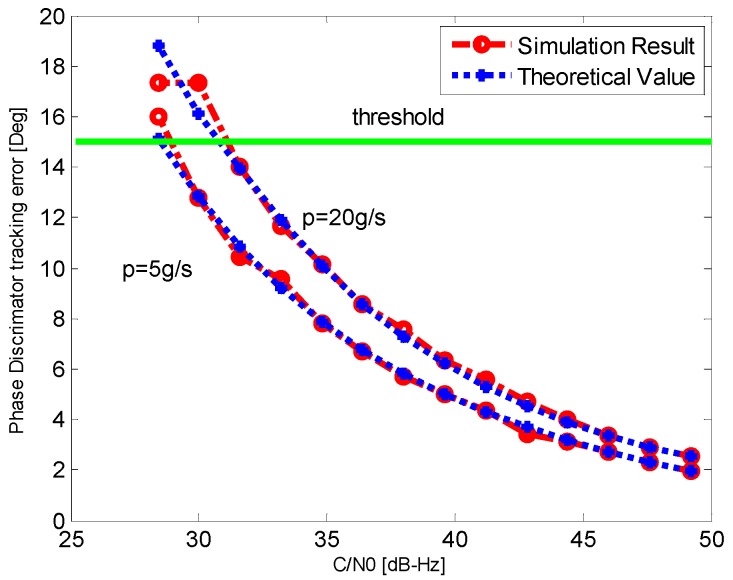
The PD tracking error for different *C*/*N*_0_.

In order to verify the validity of the adaptive adjustments of bandwidth and integration time, the simulation result of the phase tracking error of discriminator output is compared with the theoretical minimum tracking error value. Noise is added in previous GPS IF simulation data after 15 s with a rate of 1.6 dB/s to attenuate *C*/*N*_0_, and the initial *C*/*N*_0_ is set as 49.2 dB-Hz. The corresponding phase tracking errors of discriminator output are shown in Figure 9, and the theoretical value is calculated by Equation (34).

It can be seen from the Figure 9 that the phase tracking error increases with the *C*/*N*_0_ attenuation. The phase tracking error of discriminator output is almost consistent with the theoretical minimum tracking error. It demonstrates that the adaptive INS-aided PLL tracking method can achieve the minimum tracking error for different *C*/*N*_0_ and vehicle dynamic condition by choosing the optimal bandwidth and integration time effectively.

### 5.3. Simulation Result Analysis

To evaluate the performance of the adaptive tracking method, it is compared with other four INS-aided PLL tracking methods with different loop parameter selections under the simulated harsh environment. The total five loop parameter selections are *B_n_* = 10 Hz, T = 1 ms, *B_n_* = 10 Hz, T = 20 ms, adapt-*B_n_*, T = 1 ms, adapt-*B_n_*, T = 20 ms and adapt-*B_n_*, adapt-T. The performance evaluation method used in here is based on the phase discriminator output and the phase lock indicator (PLI) which can be calculated as [23].

(35) $$PLI_{n}=\frac{I_{n}^{2}-Q_{n}^{2}}{I_{n}^{2}+Q_{n}^{2}}$$

where *PLI_n_* presents the PLI value computed from correlation values $I_{n}^{2}$ and $Q_{n}^{2}$ corresponding to the *n*th tracking loop update, *PLI_n_* is averaged over 1 second to obtain more reliable estimate of the PLI.

**Figure 10:**
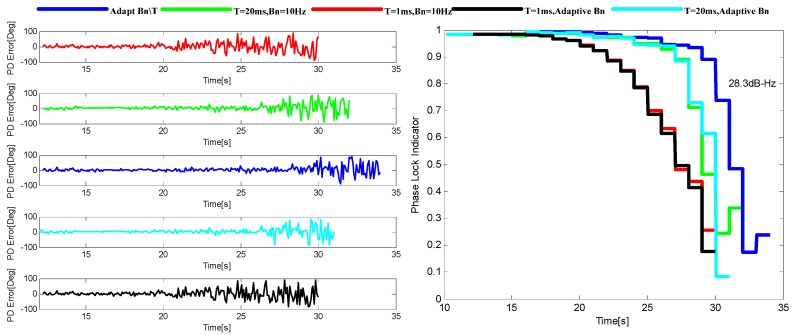
The output of PD (**left**) and the corresponding PLI (**right**) when *p* = 50 m/s^3^.

**Figure 11:**
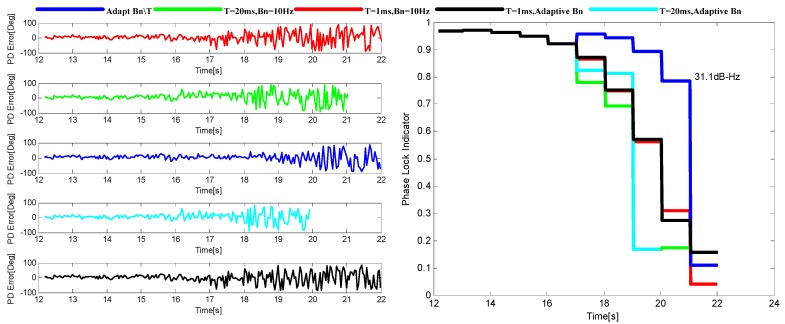
The output of PD (**left**) and the corresponding PLI (**right**) when *p* = 200 m/s^3^.

During the simulation, noise is added into the lower dynamic data with a rate of 1.5 dB/s, and a 3 dB/s noise is added into the higher one after 14 s. Figure 10 and Figure 11 show the variation of tracking performance under the two environments stated above, respectively. The adaptive tracking method achieves the best performance in both harsh environments with a 4–9 dB sensitivity improvement. The tracking methods with the 20 ms integration time show better performance than those with the 1 ms integration time when the dynamics are relatively lower, while their performance degrades more quickly when the dynamics increase. Both figures also demonstrate that the improvement by adapt-T is more significant than that by adapt-*B_n_*.

**Figure 12:**
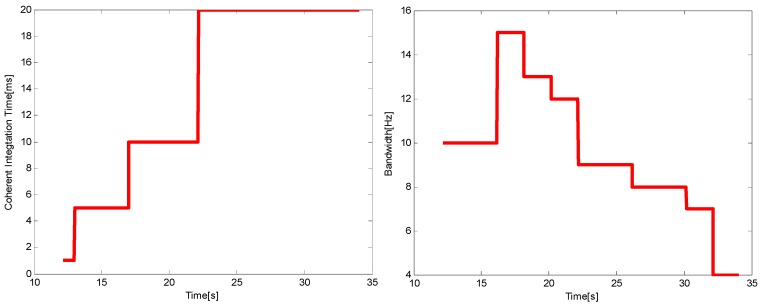
The choices of *T* (**left**) and *B_n_* (**right**) when *p* = 50 m/s^3^.

**Figure 13:**
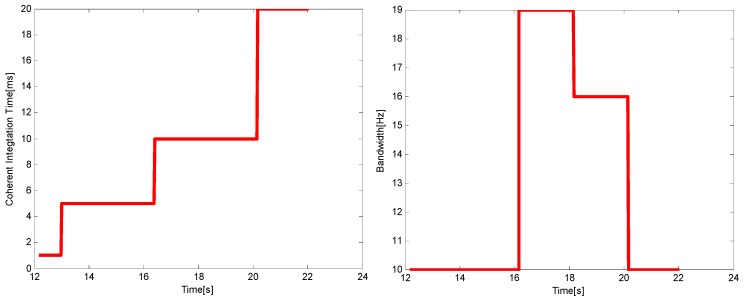
The choices of *T* (**left**) and *B_n_* (**right**) when *p* = 200 m/s^3^.

The choices of noise bandwidth and integration time under the two harsh environments are shown in Figure 12 and Figure 13, respectively. Using the adaptive tracking method, the integration time will increase with the attenuation of *C*/*N*_0_, while just the opposite occurs with the noise bandwidth. From the comparison of the two figures, it can be seen that in the higher dynamic environment, the noise bandwidth cannot be chosen too small and the choice of the integration time should be relatively short. The choices of the two parameters are in accord with the theoretical analysis results in Section 4.

Furthermore, a comparison is also made with other methods to evaluate the performance of the adaptive method. For the harsh environment with high dynamics and signal attenuation, the improvement methods for INS-aided PLL mainly focus on adjusting the noise bandwidth as in [8,15]. The main ideas of the adaptive method proposed in this paper and the methods presented in [8,15] are listed in Table 1 as follows.

**Table 1:** The comparison of three improvement tracking methods for INS-aided PLL.

Method	Main Idea
Adaptive Method	Choose the optimal integration time and bandwidth for a given application under the minimum tracking error criterion.
Method 1 ([15])	The Doppler aiding error resulting from the navigation uncertainty is derived and used to design the adaptive loop filter, so as to adjust the noise bandwidth.
Method 2 ([8])	Using the fuzzy logic controller (FLC) to adjust the PLL noise bandwidth on-line.

**Figure 14:**
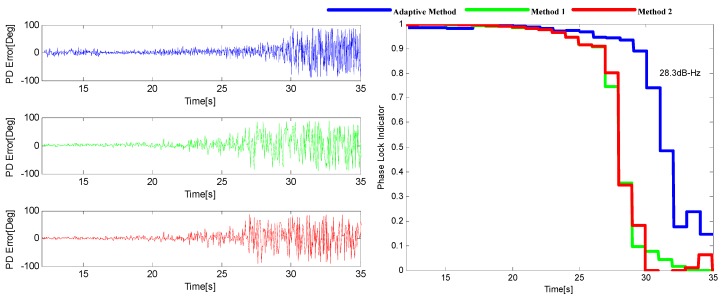
The output of PD (**left**) and the corresponding PLI (**right**) when *p* = 50 m/s^3^.

**Figure 15:**
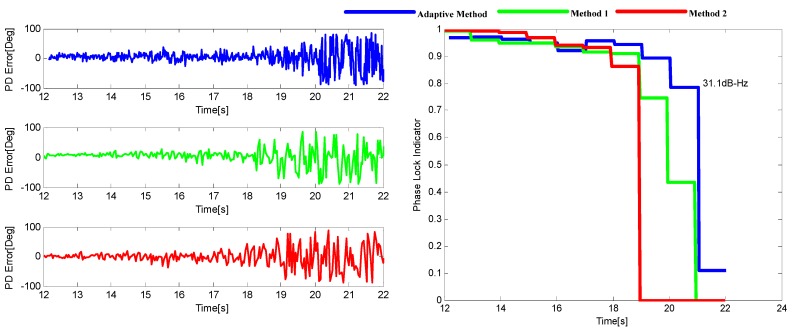
The output of PD (**left**) and the corresponding PLI (**right**) when *p* = 200 m/s^3^.

The tracking performance of these three methods under the two environments stated above are illustrated in Figure 14 and Figure 15. It can be seen that the adaptive method by choosing the optimal noise bandwidth and integration time can achieve a better performance over the other two methods that only choose the adaptive bandwidth with a 3–6 dB sensitivity improvement.

In summary, the adaptive tracking method can choose the optimal noise bandwidth and integration time under the minimum tracking error criterion. By comparing with the traditional INS-aided PLL tracking method as well as the methods of adjusting the noise bandwidth, it is seen that it works better in harsh environments. The improvement of the tracking performance can be verified by software simulation.

## 6. Hardware Simulation

**Figure 16:**
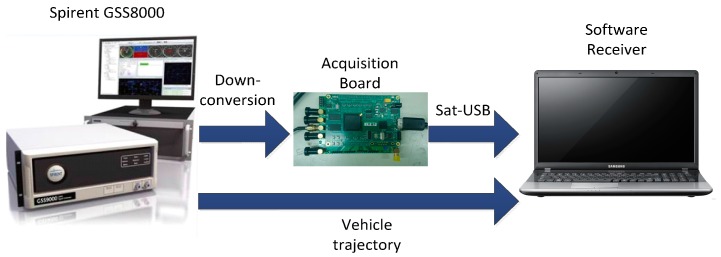
Hardware simulation testing platform.

To further test the performance of the adaptive tracking method, a hardware simulation testing platform is set up, and the overall testing platform is shown in Figure 16. The Spirent GSS8000 simulator [24,25] is used to generate the GPS L1 signal. GPS IF data is collected by an acquisition board, whose front-end bandwidth is chosen as 40 MHz, and it is driven by the TCXO referred above. The sampling frequency is chosen as 24 MHz. The simulator also provides a reference PVAT solution which can be used to derive true acceleration and angular velocity measurements through an inverse mechanization. The simulated biases of gyro and accelerometer are $\delta \omega_{ib}^{b}=10^{{}^{\circ}}/h$ and $\delta f_{ib}^{b}=1\;\text{mg}$, respectively. The output frequencies of the inertial sensor and GPS software receiver are 100 Hz and 25 Hz, respectively.

Two trajectories have been simulated for the performance test: Trajectory 1 has a relatively lower dynamic of 10 g maximum acceleration, while Trajectory 2 has a higher dynamic of 50 g maximum acceleration and 20 g/s maximum jerk. Two other improved INS-aided PLL tracking methods presented in [8,15] which are mentioned before are compared with the proposed adaptive tracking method.

### 6.1. The Test for the Trajectory with Lower Dynamic Condition

**Figure 17:**
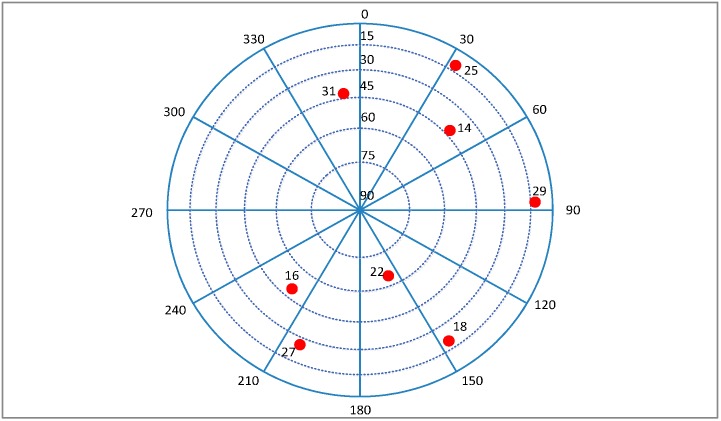
Sky plot of the satellites in Trajectory 1.

**Figure 18:**
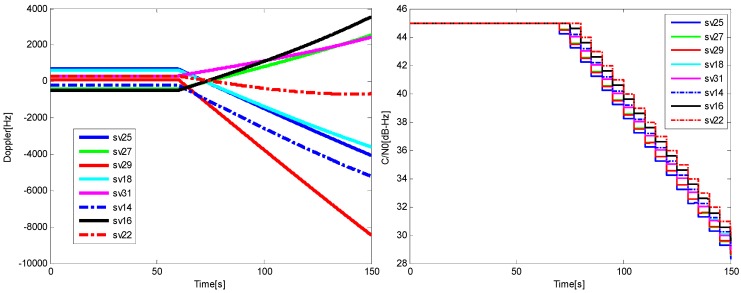
The Doppler (**left**) and *C*/*N*_0_ **(right)** for the satellites in Trajectory 1.

The vehicle is stationary at the beginning, a transient jerk is added to keep the vehicle with a linear motion of 10 g acceleration heading east after 60 s, and a noise is added into the GPS signal with a rate of 0.2 dB/s at the same time. The sky plot is shown in Figure 17, and the Doppler and *C*/*N*_0_ variations of the visible satellites are shown in Figure 18.

**Figure 19:**
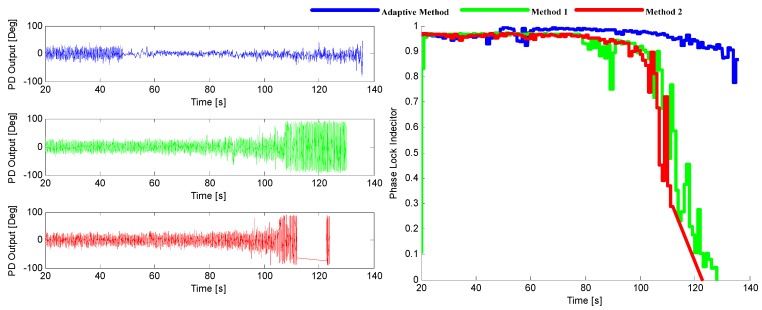
The output of PD (**left**) and the corresponding PLI (**right**) for SV29 in Trajectory 1.

Three INS-aided PLL tracking methods are tested and compared here, which are the adaptive method proposed in this paper, and the two methods presented in [8,15], respectively. Figure 19 shows the tracking performance of SV29. It can be shown that the adaptive INS-aided PLL tracking method has a much better tracking performance than the others with the tracking sensitivity increased by about 3.2 dB.

**Figure 20:**
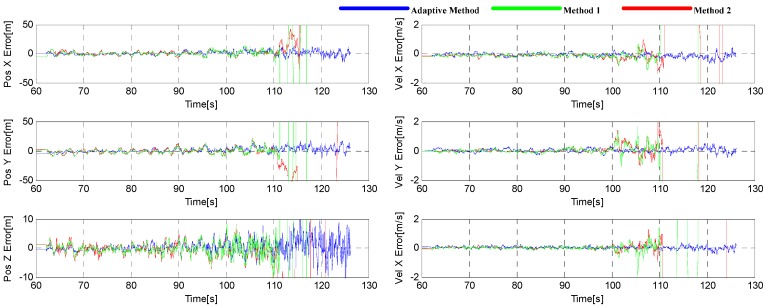
Position (**left**) and velocity (**right**) errors during the simulation in Trajectory 1.

The root mean square (RMS) of three-dimensional position and velocity error is adopted for the navigation performance comparison in this paper. The position and velocity errors are shown in Figure 20. It can be seen that the adaptive tracking method has the best performance, and a same conclusion can be also drawn from Table 2, where the RMSs of the position and velocity errors are given. From the table, we can see that the velocity error is decreased by 37% and 40%, and the position error decreased by 24% and 17%.

**Table 2:** RMSs of the position and velocity errors in Trajectory 1.

	Position Error (m)			Velocity Error (m/s)		
Method	Adaptive Method	Method 1	Method 2	Adaptive Method	Method 1	Method 2
RMS	4.9493	6.4867	5.9646	0.1926	0.3034	0.3227

### 6.2. The Test for the Trajectory with Higher Dynamic Condition

**Figure 21:**
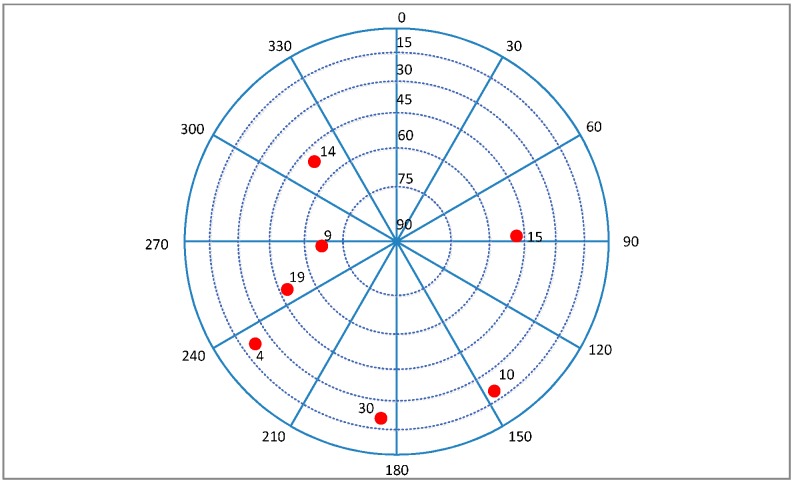
Sky plot of the satellites in Trajectory 2.

**Figure 22:**
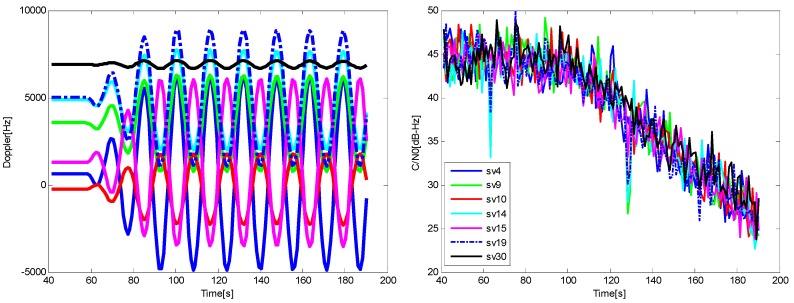
The Doppler (**left**) and *C*/*N*_0_ for satellites (**right**) in Trajectory 2.

For the higher dynamics scenario, the velocity, acceleration and jerk of the vehicle increase gradually after a 60 s stationary period, and each of them changes with time in a sine form. After ninety seconds, the amplitudes of the acceleration and jerk reach the stable maximums of 50 g and 20 g/s, respectively, meanwhile the same noise as the Trajectory 1 is added into the GPS IF data. The sky plot is shown in Figure 21, and the Doppler and *C*/*N*_0_ variations of the satellites are shown in Figure 22. 

**Figure 23:**
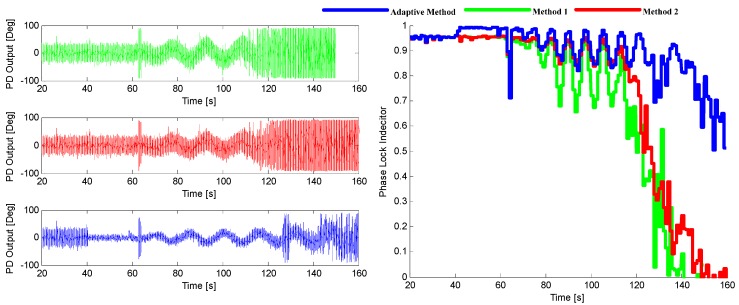
The output of PD (**left**) and the corresponding PLI (**right**) for SV10 in Trajectory 2.

The sinusoidal variation of Doppler means there are not only acceleration and jerk, but also much higher order dynamics in this scenario. The tracking performance of SV10 is shown in Figure 23, from which it can be seen that the tracking sensitivity of the adaptive method is increased about 5 dB compared with the other two methods. The result is consistent with that of the software simulation.

**Figure 24:**
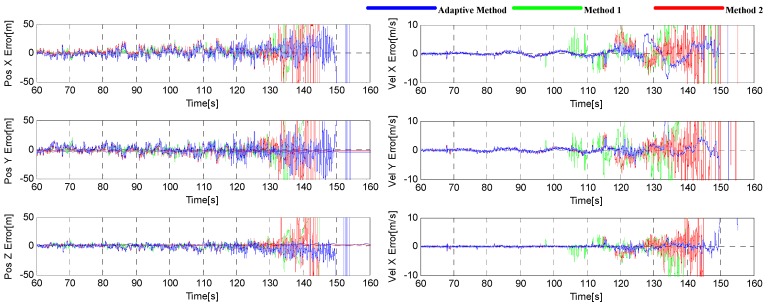
Position (**left**) and velocity (**right**) errors during the simulation in Trajectory 2.

The position and velocity errors are shown in Figure 24, and their RMSs are given in Table 3. We can see that the navigation performance of the adaptive tracking method is also better than others, with the velocity error decreased by 50% and 36%, and position error decreased by 6% and 7%.

**Table 3:** RMSs of the position and velocity errors in Trajectory 2.

	Position Error (m)			Velocity Error (m/s)		
Method	Adaptive Method	Method 1	Method 2	Adaptive Method	Method 1	Method 2
RMS	8.9449	9.5028	9.5726	0.9431	1.9048	1.4951

In summary, the hardware simulation results demonstrate that the adaptive INS-aided PLL tracking method can improve the tracking ability and navigation performance significantly under harsh environment, even the environment with complex high dynamic and signal attenuation.

## 7. Field Test

**Figure 25:**
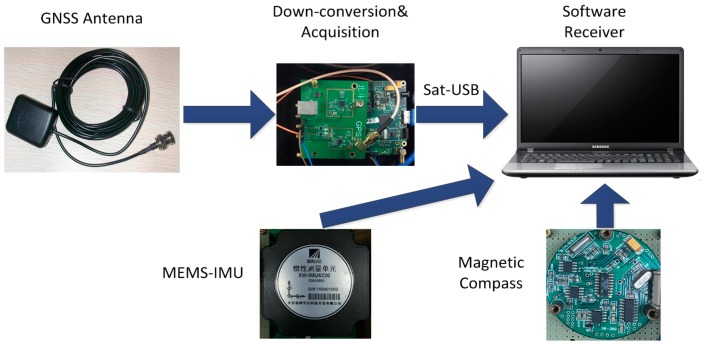
Experimental setup used for the field test.

A field test in an urban area was conducted to validate the simulation results obtained in the previous sections, and to evaluate the adaptive tracking method in the actual environment. The test system consists of a GNSS antenna connected to an acquisition board, a MEMS-IMU and a magnetic compass for initial alignment. The acquired data are post-processed in the laboratory using the adaptive tracking method to derive a navigation solution. The experimental setup used is shown in Figure 25.

**Figure 26:**
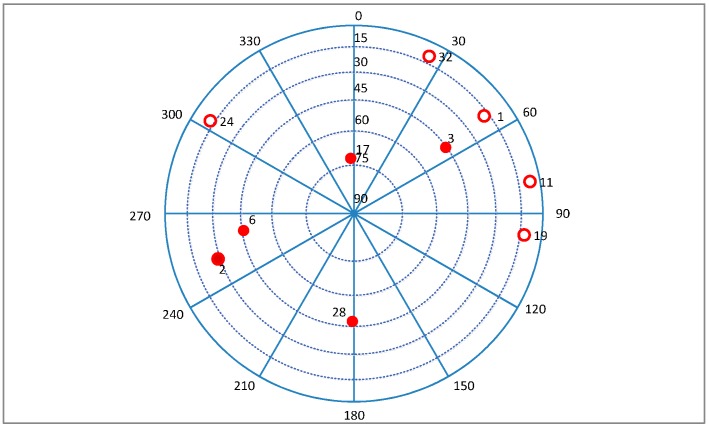
Sky plot of the satellites in field test.

The MEMS-IMU is the XW-IMU5220 from Beijing Starneto Technology Co. Ltd, (Beijing, China). The bias stability of gyro and accelerometer are 0.02°/s and 8 mg, respectively. The output frequency is 100 Hz. The measurement precision of magnetic compass is 1.5°. GPS IF data is collected by an acquisition board, whose front-end bandwidth is 40 MHz, and it is driven by the TCXO referred above. The sampling frequency is 12 MHz. The experimental site is on the east side of Datun Road, in the Beijing Olympic Park. The car moves along a rectangular block for about 7 min. The sky plot is shown in Figure 26. The number of visible satellites changes frequently due to the blockage and attenuation of surrounding buildings and trees. The satellites 19, 11, 1, 32, and 24 are not always available during the experiment.

**Figure 27:**
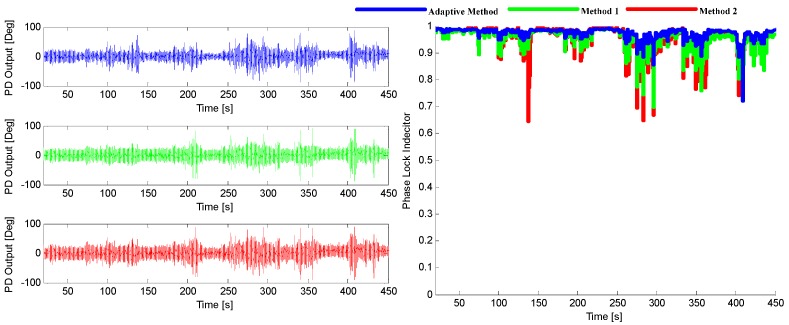
The output of PD (l**eft**) and the corresponding PLI (**right**) for SV6.

**Figure 28:**
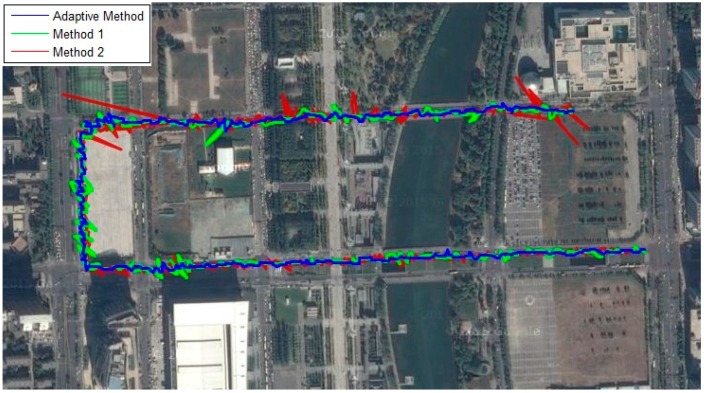
Position solutions during the field test.

Three tracking methods that are referred in Section 5 and Section 6 are tested and compared in this part, which are adaptive method and the methods presented in [8,15], respectively. The tracking performance of SV6 of these three methods is illustrated in Figure 27, and the position solutions are shown in Figure 28. It can be seen that the tracking quality and positioning accuracy of the adaptive method are better than the other two methods.

In summary, the experimental results obtained from the field test validate the simulation results described in the previous sections. It can be seen that in a complex urban environment, the adaptive INS-aided PLL tracking method shows better accuracy and stability than the methods of adjusting the noise bandwidth presented in [8,15].

## 8. Conclusions

In order to improve the adaptability of GNSS carrier tracking for harsh environments with high dynamics and signal attenuation, an adaptive INS-aided PLL tracking method was proposed. Based on the theoretical analysis of the INS-aided PLL tracking error in the discrete-time domain, both the noise bandwidth and the integration time of INS-aided PLL can be adjusted adaptively under the minimum tracking error criterion. Software simulation results demonstrate that the adaptive INS-aided PLL tracking method can achieve the theoretical minimum tracking error for the harsh environment by choosing the optimal bandwidth and integration time. Hardware simulation results show that the adaptive tracking method can effectively improve the PLL tracking ability and GNSS/INS integrated navigation performance. For harsh environments, the tracking sensitivity is increased by 3 to 5 dB, velocity error is decreased by 36% to 50% and position error is decreased by 6% to 24% when compared with the INS-aided PLL method of adjusting the noise bandwidth. Finally, the adaptive method is evaluated in a field test in an urban environment with low-quality MEMS inertial sensors. The measurements obtained are in accordance with the simulation results and demonstrate the tracking performance of the method. Therefore, the adaptive INS-aided PLL tracking method can offer a superior performance for the GNSS receiver under harsh environment conditions.

## References

[1] El-Rabbany A. (2002). Introduction to GPS: The Global Positioning System.

[2] Kaplan E., Hegarty C. (2005). Understanding GPS: Principles and Applications.

[3] Wang X., Ji X., Feng S., Calmettes V. (2015). A high-sensitivity GPS receiver carrier-tracking loop design for high-dynamic applications. GPS Solut..

[4] Li W., Liu S., Zhou C., Zhou S., Wang T. High Dynamic Carrier Tracking Using Kalman Filter Aided Phase-Lock Loop. Proceedings of the International Conference on Wireless Communications, Networking and Mobile Computing (WiCom 2007).

[5] Han H., Wang J., Wang J., Tan X. (2015). Performance Analysis on Carrier Phase-Based Tightly-Coupled GPS/BDS/INS Integration in GNSS Degraded and Denied Environments. Sensors.

[6] O’Driscoll C., Petovello M.G., Lachapelle G. (2011). Choosing the coherent integration time for Kalman filter-based carrier-phase tracking of GNSS signals. GPS Solut..

[7] Petovello M., Sun D., Lachapelle G., Cannon M. Performance analysis of an ultra-tightly integrated GPS and reduced IMU system. Proceedings of the 20th International Technical Meeting of the Satellite Division of The Institute of Navigation ION GNSS.

[8] Qin H., Sun X., Cong L. (2013). Using fuzzy logic control for the robust carrier tracking loop in a Global Positioning System/Inertial Navigation System tightly integrated system. Trans. Inst. Meas. Control.

[9] Qin F., Zhan X., Du G. (2013). Performance Improvement of Receivers Based on Ultra-Tight Integration in GNSS-Challenged Environments. Sensors.

[10] Razavi A., Gebre-Egziabher D., Akos D.M. (2008). Carrier loop architectures for tracking weak GPS signals. IEEE Trans. Aerosp. Electron. Syst..

[11] Alban S., Akos D., Rock S.M., Gebre-Egziabher D. Performance analysis and architectures for INS-Aided GPS tracking loops. Proceedings of the 2003 National Technical Meeting of The Institute of Navigation 2003.

[12] Gao G., Lachapelle G. INS-assisted high sensitivity GPS receivers for degraded signal navigation. Proceedings of the 19th International Technical Meeting of the Satellite Division of The Institute of Navigation.

[13] Gebre-Egziabher D., Razavi A., Enge P.K., Gautier J., Pullen S., Pervan B.S., Akos D.M. (2005). Sensitivity and Performance Analysis of Doppler-Aided GPS Carrier-Tracking loops. Navigation.

[14] Lian P. (2004). Improving Tracking Performance of PLL in High Dynamic Applications. Master Thesis.

[15] Sun D., Petovello M., Cannon M. (2010). Use of a reduced IMU to aid a GPS receiver with adaptive tracking loops for land vehicle navigation. GPS Solut..

[16] Patapoutian A. (1999). On phase-locked loops and Kalman filters. IEEE Trans. Commun..

[17] Pany T., Riedl B., Winkel J., Wörz T., Schweikert R., Niedermeier H., Lagrasta S., López-Risueño G., Jiménez-Baños D. (2009). Coherent integration time: The longer, the better. Inside GNSS.

[18] Tsujii T., Fujiwara T., Suganuma Y., Tomita H., Petrovski I. (2011). Development of Ins-aided GPS tracking loop and flight test evaluation. SICE J. Control Meas. Syst. Integr..

[19] Tawk Y., Tomé P., Botteron C., Stebler Y., Farine P.-A. (2014). Implementation and performance of a GPS/INS tightly coupled assisted pll architecture using MEMS inertial sensors. Sensors.

[20] Zhuang W. (1996). Performance analysis of GPS carrier phase observable. IEEE Trans. Aerosp. Electron. Syst..

[21] Stephens S., Thomas J. (1995). Controlled-root formulation for digital phase-locked loops. IEEE Trans. Aerosp. Electron. Syst..

[22] Irsigler M., Eissfeller B. (2002). PLL tracking performance in the presence of oscillator phase noise. GPS Solut..

[23] Bhaskar S. Exploiting quasi-periodicity in receiver dynamics to enhance GNSS carrier phase tracking. Proceedings of the 27th International Technical Meeting of the Satellite Division of the Institute of Navigation 2014.

[24] Jovanovic A., Tawk Y., Botteron C., Farine P.-A. Multipath mitigation techniques for CBOC, TMBOC and ALTBOC signals using advanced correlators architectures. Proceedings of the 2010 IEEE/ION Position Location and Navigation Symposium (PLANS).

[25] GSS8000 Multi-GNSS Constellation Simulator. http://www.spirent.com/Solutions-Directory/GSS8000.

